# Stricture of the Urethra, with Retention of the Urine; Division of the Stricture; Puncture of the Bladder; Recovery

**Published:** 1846-06

**Authors:** William Caldwell

**Affiliations:** Louisville, Ky.


Art. II.—*Stricture of the Urethra, with Retention of the Urine; Division of the Stricture; Puncture of the Bladder; Recovery.* By William Caldwell, M.D., of Louisville, Ky.

Mr. R. M. W.——, aged 24 years, of small stature, and bilious temperament, with black hair and large prominent grey eyes, had been generally healthy, until he contracted gonorrhœa in November, 1835, for which he was treated with emetics, Cooke's pills, balsam of copaiva, and nitre. The discharge from the urethra was several times checked, but as often returned again, until February, 1836, when he was seized with retention of urine. This was partially relieved by the use of fomentations, &c., but from that time he continued to have difficulty in micturition, accompanied with more or less gleet. In June following he was attacked with complete retention; his physician attempted to introduce the catheter, but failed; and he again found partial relief, after twelve hours, in the use of nauseants, venesection, diuretics, and fomentations.

From June, 1836, he had more or less difficulty in micturition, but no complete retention, and was under no medical treatment, until the fall of 1843, when he again contracted gonorrhœa and was treated with Chapman's mixture. At the end of six weeks he was dismissed as cured, but was still the subject of stricture accompanied by gleet.

On the 16th of June, 1844, I was called, for the first time, to see him, in company with Dr. Field. We found the bladder greatly distended from complete retention.

In presence of Dr. Field, I attempted to introduce a catheter, but failed; some resistance was met with just within the scrotum, but it yielded readily, and about an inch further on the instrument encountered a resistance so firm and unyielding that I became satisfied that it rested upon an organized stricture. Finding it impossible to introduce an instrument, we resorted to copious venesection, and other means, to produce relaxation, which in a short time afforded him partial relief. Again on the 24th of July, I was called to see him; found him laboring under complete retention, and relieved him as before. I then advised an operation as the only means of effecting a permanent cure, but he objected to it. He subsequently placed himself under several physicians at different times for treatment; and I saw him no more professionally until about the first of December following, when I was called to see him in consultation with Dr. Squires. He was again laboring under complete retention. I made another ineffectual effort to introduce the catheter; and he was partially relieved as previously. He had now been constantly under the influence of constitutional treatment with strict diet since July, which had only served to prevent complete retention, having made no appreciable progress towards removing the stricture. His constitutional treatment with restricted diet was continued, for the purpose of keeping off if possible another attack of retention, until he could place himself under surgical treatment.

On the 22d of April, 1845, I was called to see him, and found him suffering excruciating pain with distended bladder.

I again attempted to introduce the catheter but failed. Every effort made by myself and others, when called upon to examine his case, to pass instruments of different kinds into the bladder, having failed, any hope of succeeding seemed to be absurd.

Deeming an operation the only resource left to save the patient's life, and afford him permanent relief, I sent for my partner, Dr. John D. Winston, of Columbia, Ky., at which place I then resided. Dr. W. attempted to introduce an instrument, but without success; and agreed with me that it was impracticable, and that the operation should be performed.

We accordingly explained to the patient and his friends the nature and danger of the operation; that it was probable the incision would have to be continued to the bladder, and the wall of the bladder punctured, forming a new canal and neck to that viscus; and that the operation was condemned by high authority, &c. But he called upon me to operate; stating that for nine years he had not voided his urine without pain; that at best it merely dribbled away between his feet; and that he was evidently growing worse, so much so that his life had become a burthen to him.

With the concurrence and assistance of Dr. Winston, I determined to proceed to the operation the next morning.

April 23d. We proceeded, in the presence of Drs. Field and Hardin, to the operation. Our patient was placed on a common dining table on his back, confined, as in the operation for stone, with his legs flexed, and his heels resting upon the palms of his hands; a silver catheter was passed into the urethra until it rested upon the stricture, and was then given to the hands of an assistant. Standing between the legs of the patient, at the foot of the table, I cut down upon the catheter behind the scrotum; a grooved director was then passed into the urethra and upon it the incision was continued through the stricture; the walls of the urethra at this point were very dense and firm, and about an eighth of an inch thick. With some difficulty, the catheter was carried forwards about three-fourths of an inch, when it was again arrested; the director was then resorted to, and the canal was fairly laid open through the second stricture. The parietes of the canal at this point were about a quarter of an inch thick, and of a semi-cartilaginous appearance. Thus far the operation progressed favorably, no obstacle having presented itself which was not readily overcome. We had now a cut externally of two inches, and the urethra divided to the extent of an inch and three-quarters from the bulb backwards towards the bladder. But in attempting to trace the canal further we found the end of the director resting on the ulcerated parts, which seemed to have no communication with the membranous portion of the canal. Having failed in every effort with the director, a small probe, and the catheter, to trace the canal further, we directed the patient to pass his urine, hoping to find the canal by tracing the stream of urine; but in this we failed also, for it seemed to force its way into the wound through the diseased and mutilated parts in every direction, giving no clue to the natural channel, or any communication with the bladder whatever. Our patient being greatly exhausted, it was determined to defer any further search until he should have some rest; accordingly we placed him in bed where he spent the remainder of the day and night in tolerable comfort.

24th. During the day, when the patient's strength would permit, every effort was made to trace the canal, until late in the evening, when retention came on, and we gave over the search. It was then agreed that we should continue the operation, but it was thought best to defer it until morning, because it was then late, the bladder was not as full as we wished it, and the patient was very much fatigued; at the same time there was no indication calling for immediate interference. He spent a bad night.

25th. The mode and character of operation being agreed upon, we proceeded, in the presence of Drs. Fields, White, and Walker, to continue the incision into the bladder, puncturing the wall of this organ as near the neck as possible. The patient being placed on the table and confined as previously, the fore finger of the left hand was passed into the rectum and slightly depressed. A sharp-pointed bistoury was then seized between the thumb and forefinger of the right hand and carried into the wound with the point resting on the forefinger. Thus guarded, as the way was felt by the finger, the point of the instrument was carried forward, and with lateral motions given it by the thumb, the parts were divided to the bladder, which was punctured with a free incision. As the urine gushed out a female catheter was inserted from the external wound into the bladder, and the end stopped; a male catheter was then inserted into the urethra, and, the other being withdrawn, was conveyed through the newly formed canal to the cavity of the bladder. It was then confined in its place, the external wound dressed, and the patient returned to bed. During the afternoon, doing well; 7 o'clock, P.M., had a slight chill—lasting 10 minutes, followed by fever; pulse 130; distressing thirst; gave solution of cream of tartar. Nine o'clock at night—sweating; pulse reduced; feels comfortable. Twelve o'clock—severe chill; great distress, with difficulty of breathing; ordered calomel and morphine; mustard sinapisms to the spine, with warm applications to the extremities; chill lasted half an hour, followed by fever, thirst, &c.; pulse fleeting. Two o'clock—commenced sweating; pulse very quick.

26th. *Morning*—Improved in general symptoms; quiet, with moist skin. ℞. Calomel and morphine every five hours, and elm mucilage as a drink. Ten o'clock—pulse 127; skin moist; urine passes freely through the catheter, and also through the wound. Twelve o'clock—slight chill. ℞. morphine $\frac{1}{4}$gr. Chill followed by slight fever and sweating; had a restless night.

27th. Some improvement; pulse 117; skin moist; urine passes freely as before; some appetite; suspend medicines. Ordered milk and rice diet; mucilaginous drinks.

28th. Still improving; no action from the bowels since the 25th; gave enema. Five o'clock, P.M., pulse 95; still improving, but no action from the bowels; repeat enema; spent a restless night.

29th. Pulse and general condition about the same. Two o'clock, P.M., catheter clogged up; withdrew it and inserted a female catheter through the incision in the perineum; having prepared a male catheter it was passed into the urethra, and, after withdrawal of the other, was carried on to the bladder without difficulty; the external wound was closed and dressed with adhesive strips. Spent a comfortable night.

30th. Urine passes freely through the catheter with some bloody matter; pulse 95. Ordered thin squirrel soup as diet.

May 1st. Skin pleasant; no thirst; pulse 88; appetite good; tongue clean and healthy. Ordered enema; chicken soup as diet. Five o'clock, P.M.—enema brought away healthy discharge from the bowels; pulse 85; doing well.

2d. Still improving; pulse 80; tongue clean and moist; appetite good; rested well since yesterday. Ordered milk and farinaceous diet.

From this time there was gradual and steady improvement. The catheter was examined daily, but the parts seemed to be healing so kindly and were so free from irritation, that it was thought best not to remove it until the 3d of June, when it was removed and cleansed, and re-inserted without difficulty; the patient exhibiting very little tenderness. On the 6th, the catheter was removed at 10 o'clock, A.M., and was not replaced until 5 o'clock, P.M. The urine passed freely; there was no pain or tenderness; the parts felt natural.

On the 8th, the instrument was removed and not replaced until the next day. The urine passed in a full, free stream; parts feeling perfectly sound, and the patient's health and strength daily improving.

From this time, the period for leaving out the catheter was gradually lengthened. I saw the patient occasionally until he learned to remove and introduce the instrument himself without difficulty. He was then dismissed as cured, with the direction to introduce the catheter two or three times a week for some months, by way of precaution.

I saw him in January, 1846; he was perfectly well, had entire control over the urinary organs, and voided urine in a full, free stream, with no sensation but that experienced by a man in perfect health.

April, 1846.

